# Social, economic, and legislative factors and global road traffic fatalities

**DOI:** 10.1186/s12889-020-09491-x

**Published:** 2020-09-17

**Authors:** Mohammad Reza Rahmanian Haghighi, Mohammad Sayari, Sulmaz Ghahramani, Kamran Bagheri Lankarani

**Affiliations:** Health Policy Research Center, Institute of Health, Shiraz University of Medical Sciences, Shiraz, Iran

**Keywords:** Human development index, Education, Income, Life expectancy, GINI index, Road traffic fatalities

## Abstract

**Background:**

Road traffic fatalities (RTF) is the 8th cause of mortality around the world. At the end of the Decade of Action, it would be of utmost importance to revisit our knowledge on the determinants of RTF. The aim of this study is to assess factors related to RTF at global level.

**Methods:**

We used road safety development index which accounts for the interactions between system, human and products to assess the RTF in 115 and 113 countries in 2013 and 2016, respectively. To analyze data, three statistical procedures (linear regression, classification and regression trees, and multivariate adaptive regression splines) were employed.

**Results:**

Classification and regression trees has the best performance amongst all others followed by multivariate adaptive regression splines for 2013 and 2016 data set with an R^2^ around 0.83. Results show that any increase in human development index was associated with RTF reduction. Comparing RTF data of 2013 and 2016, 8 countries experienced a change of more than 30%, which demonstrated a significant relationship with GINI index (named after Corrado Gini). Considering the three components of human development index, it is revealed that education explained most of RTF variation in classification and regression trees model followed by income and life expectancy.

**Conclusion:**

Policymakers should consider road collisions as a socio-economic issue. In this regard, they can make provisions to reduce RTF in the long run by focusing on enhancing the three components of human development index, mainly education. However, there is a need to investigate the causation pathway among these three components with RTF with different time-trend procedures.

## Background

According to recent World Health Organization (WHO) reports, road traffic fatality (RTF) was the 8th leading cause of death worldwide in 2016 [1]. In this year, approximately 1.35 million people were killed in road collision. Based on WHO’s report (2018), A total of 40% of motorized vehicle are registered in high-income countries (with Gross National Income (GNI) per capita of more than 12,235 current US dollar), but only 7% of RTF has occurred in these countries. Middle-income (with GNI per capita between 1006 and 12,235 current US dollar), and low-income (with GNI per capita of less than 1006 current US dollar), countries with 59 and 1% registered vehicles have 80 and 13% of RTF, respectively [2].

Infrastructures such as land-usage planning, road layout, designing for road function and vehicle safety are important factors in preventing RTF but too much focus has been devoted on road users’ characteristics and behaviors [3]. Most studies were conducted at micro levels on these factors, reporting the effect of road users’ age, level of education, gender, drug and alcohol consumption, fatigue and other factors [3–5]. Research on social aspects of road collision at community level have shown that low socioeconomic status is associated with higher RTF as people living in deprived areas are more exposed to road traffic injuries and receive less efficient care [6, 7]. Confounding factors such as the use of older vehicles and vehicles with lower safety conditions might also exacerbate the situation. Vulnerable groups such as pedestrians, cyclists and motorcyclists which consist nearly half of the victims in road collisions are also more concentrated in deprived neighborhoods [8].

The perspective about road collision has changed in the last century from focusing only on individual behaviors to the importance of supportive environment. It was recognized that individual users do not have the ability to reduce the risk of collisions and governments have to play a more active role in reducing road collisions through legislative processes and various forms of regulation [9].

RTF should be approached as a social problem within the context of each individual community [10]. This would affect the priorities in interventions and legislations which may differ between and within countries.

Nowadays, there is more emphasis on the interaction between person, machine and environment in accident [11]. Haddon work recognizing the importance of the whole area of “mishaps” with all extra-rational conditions in accidents should be considered a turning point as he proposed a systematic approach rather than telescopic interventions in reducing RTF [12]. Based on Haddon’s approach, the major source of errors can be identified, and appropriate interventions can be utilized to reduce the risk of accidents or to mitigate the adverse effects of accidents including crash severity, fatalities and injuries [13].

Along the same line, Al-Haji (2007) proposed road safety development index (RSDI) as a comprehensive conceptual framework to approach road collisions. In RSDI, three dimensions of performance, which include human, product and system performance are considered. He considered the system as a whole with the interactions between system, human and products. Accordingly, “system performance” would be the effectiveness of interventions in improving car safety, safer roads and safer use. Table 1 summarizes factors based on RSDI [10].

**Table 1:** Road Safety Development Index (RSDI) Factors [10]

RSDI components	Factors
Human performance	• Safer road user’s “behavior”
Product performance	• Percentage change of death trend • Personal risk “death per population” • Traffic risk “death per vehicle”
System performance	• Safer roads • Safer vehicles • Enforcement performance • Organizational performance • Socioeconomic performance

At the end of the Decade of Action, it is crucial to assess factors related to RTF. Most studies considered limited countries or factors, but scanty research has been carried out on the comparison between the less developed countries and high developed countries in this respect. Regarding these limitations, this study aimed to investigate the main factors associated with RTF at global level and accordingly, we adopted three different analytical methods to reach the best explanation.

## Methods

### Data sources and indices

We used the latest published data for safer roads and mobility, safer vehicles, and safer road users in the “Global status report on road safety 2015” and “Global status report on road safety 2018”, published by WHO. The data of 180 countries were collected in 2013 and 2016 reports.

After list-wise deletion (eliminating samples with any missing values), 115 and 113 countries were selected from 2015 and 2018 reports, respectively. To consider different dimensions of the system performance, three aspects of safe system were chosen (safer roads and mobility, safer vehicles, and safer road users) based on WHO’s reports for each country in the target years. The selected indices for each factor are available in Additional file 1: Table S1. Data on organizational performance (one of the RSDI components) was not available. “Death per population” was chosen for product performance. Data as “death per population” and “death per vehicles” factors were analyzed to obtain a more holistic view on the outcome.

Human Development Index (HDI) incorporated three major indices of life expectancy, GNI and GINI (named after Corrado Gini). Life expectancy refers to a long and healthy life, while education and GNI signify access to knowledge and decent standard of living, respectively. Regarding the GINI index, it is defined by the World Bank as the distribution of income (or, in some cases, consumption expenditure) among individuals or households within an economy [14]. Urban population and unemployment rate are other factors extracted from the United Nations Development Program and World Bank data center, with the former defined by the World Bank as “people living in urban areas as defined by national statistical offices” (per 100,000) [15] and the latter as “the share of the labor force that is without work but available for and seeking employment” [16]. These include the socioeconomic performance indices and factors in target years. We also considered the data from world happiness (“underpinnings of measuring and understanding subjective well-being”) [17] and homicide rate (per 100,000 people) (World Bank definition: “Intentional homicides are estimates of unlawful homicides purposely inflicted”) [18] as indices of the “behavior” of safer road user using the latest data available for each country. The RSDI components, factors and indices used, and the data sources are summarized in Table 2.

**Table 2:** RSDI components, Indices and data resources

RSDI components	Selected factors	Indices	Data sources
Human performance	Safer road user’s “behavior”	Happiness	World happiness: Trends, explanations and distribution (2013) and (2016) [17, 19]
		Homicides (per 100,000 people)	World Bank [18]
Product performance	Personal risk “death per population”	Mortality caused by road traffic injury (per 100,000 people)	Global status report on road safety 2015 and 2018 [2, 8]
System performance	Safer roads		Global status report on road safety 2015 and 2018 [2, 8]
	Safer vehicles		
	Enforcement performance		
	Socioeconomic performance	HDI	UNDP [20]
		Urban population (% of total)	World Bank [15]
		GINI index (World Bank estimate)	World Bank [14]
		Unemployment, total (% of total labor force) (modeled ILO estimate)	World Bank [16]

*RSDI* Road safety development index, *ILO* International labour organization, *HDI* Human development index, *UNDP* United Nations Development Programme

### Statistical analysis

In road safety analysis, regression analysis such as linear regression models and Poisson regression has been the most conventional procedures to determine the factors affecting mortality rate. However, they have to meet certain assumptions and if we ignore the pre-defined assumptions, estimation would be inaccurate [21].

In order to overcome these limitations, other procedures such as classification and regression trees (CART) and multivariate adaptive regression splines (MARS) were applied in road safety analysis. They are considered decision tree procedures, and MARS can be viewed as the modified version of CART [22]. Acciani et al. (2011) showed that MARS and CART perform computationally well with small datasets [23]. In this research, two non-parametric statistical procedures, CART and MARS, were employed to determine and classify factors associated with mortality rate. Furthermore, the prediction indices were compared to a stepwise multivariate linear regression (SMLR).

### Classification and regression trees

Classification and regression trees is a tree-based procedure, which can be applied for continuous and discrete variables. CART algorithm uses all data sets to build child nodes by splitting the subsets of the entire predictor variables. The goal is to obtain a maximum homogeneous subset of the data with regard to the dependent variable. Especially, when the target variable is continuous, CART uses the least squared deviation (LSD) criterion to build an optimal tree. The split is chosen to maximize the value of LSD criterion function. The split procedure performs recursively into terminal nodes [24].

### Multivariate adaptive regression splines model

Multivariate adaptive regression splines is a nonparametric data mining procedure, which combines the classical linear regression, the spline functions and the recursive partitioning. It uses a set of piecewise function called basis function (BF) to determine relationships between a set of independent variables and the target variable [22, 25]. The general expression of MARS is defined as follow:

$$ \hat{y}={\beta}_0+\sum \limits_{m=1}^M{\beta}_m{h}_m(x) $$

Where $\hat{y}$ is the target variable predicted by the model, M is the number of selected BFs, *β*_0_ is the constant term, *β*_*m*_ is the coefficient of the *m*-th BF and a *h*_*m*_(*x*) (hinge function) is one or more functions defined as follow:

$$ h(x)=\max \left(0,X-t\right)=\Big\{{\displaystyle \begin{array}{c}X-t, ift<X\\ {}0, otherwise\end{array}}\operatorname{} $$

Or

$$ h(x)=\max \left(0,t-X\right)=\Big\{{\displaystyle \begin{array}{c}t-X, ifX<t\\ {}0, otherwise\end{array}}\operatorname{}. $$

The optimal model is chosen by generalized cross-validation criterion (GCV) [26].

### Prediction performance indices

In order to compare and assess the prediction performance of the models, the performance indices such as correlation coefficient ($r=\mathit{\operatorname{cov}}\left({y}_i,{\hat{y}}_i\right)/{\sigma}_{y_i}{\sigma}_{{\hat{y}}_i}$), root mean-squared error ($RMSE=\sqrt{\sum \limits_{i=1}^n{\left({\hat{y}}_i-{y}_i\right)}^2/n}$), mean absolute error ($MAE=\sum \limits_{i=1}^n\left|{\hat{y}}_i-{y}_i\right|/n$), relative absolute error ($RAE=\sum \limits_{i=1}^n\left|{\hat{y}}_i-{y}_i\right|/\sum \limits_{i=1}^n\left|{y}_i-\overline{y}\right|$) and R-squared (${R}^2=1-\left(\sum \limits_{i=1}^n{\left({\hat{y}}_i-{y}_i\right)}^2/\sum \limits_{i=1}^n{\left({y}_i-\overline{y}\right)}^2\right)$) were estimated, where *y*_*i*_ is the *i*− th actual value of the dependent variable, ${\hat{y}}_i$ is the corresponding predicted value and *n* is the number of observations [23].

## Results

### The data set

The data set includes HDI and its components (education, income and life expectancy), happiness, GINI index, urban population, unemployment, homicide, safer roads and mobility, safer vehicles and safer road users as independent variables. Using stated procedures, an initial analysis was performed found that the HDI was the most important variable amongst all independent variables (Additional file 1: Tables S2 to S8). Afterward, to identify the main factors related to RTF, HDI components have been considered in the analysis. Moreover, in order to assess the predictive capacity of the models, a 10-fold cross-validation was carried out. Table 3 presents common descriptive statistics for all the data set for 115 countries in 2013 and 113 countries in 2016. Furthermore, Tables 4 and 5 represent the frequency of countries based on human development analytical (United Nations Development Programme) and income analytical (World Bank classification schemes).

**Table 3:** Common descriptive statistics of the variables

variable	Minimum		Maximum		Range		Mean		Standard Deviation	
	2013	2016	2013	2016	2013	2016	2013	2016	2013	2016
Mortality rate^a^	2.8	2.7	36.2	35.9	33.4	33.2	16.561	16.122	9.219	9.2045
HDI	.340	.351	.946	.951	.606	.600	.7152	.7252	.156	.157
GINI index	25.4	25.0	63.4	63.0	38.0	38.0	37.457	37.078	7.902	7.4880
Homicides^b^	.183	2.905	74.28	7.526	74.096	82.559	6.489	5.351	9.944	1.1829
Happiness	2.936	.2835	7.693	82.842	4.757	4.621	5.460	6.217	1.105	10.684
Urban population^c^	15.437	12.388	97.776	97.919	82.339	85.531	58.908	59.635	20.949	21.485
Unemployment^d^	.3192	.524	28.996	26.55	28.677	26.027	8.425	7.629	6.410	5.747
Safer roads and mobility	0	.50	5	5.00	5	4.5	3.16	3.668	1.405	1.131
Safer vehicles	0	3.00	3	7.00	3	4	1.07	6.257	1.387	.998
safer road users	2	0.00	7	4.00	5	4	6.33	1.442	.915	1.817
Education	.204	.212	.941	.940	.737	.728	.66396	.67466	.178088	.181785
Income	.287	.287	.975	.984	.688	.697	.69570	.70135	.174642	.176655
Life expectancy	.468	.514	.975	.981	.507	.467	.80230	.81301	.125288	.117707

^a^road traffic injury per 100,000 people

^b^Homicides per 100,000 people

^c^% of total

^d^% of total labor force

*HDI* Human development index

**Table 4:** Frequency of countries based on Human Development analytical category

	2013					2016				
	Frequency	Percent	Mean of mortality rate	Minimum mortality rate	Maximum mortality rate	Frequency	Percent	Mean of mortality rate	Minimum mortality rate	Maximum mortality rate
Very High^a^	39	33.9	6.751	2.8	13.7	42	37.2	7.000	2.7	18.0
High^b^	28	24.3	18.614	7.7	36.2	25	22.1	18.064	6.4	34.6
Medium^c^	26	22.6	19.908	10.5	29.1	25	22.1	19.628	9.7	30.4
Low^d^	22	19.1	27.382	14.2	35.0	21	18.6	27.881	21.4	35.9
Total	115	100.0	16.561	2.8	36.2	113	100.0	16.122	2.7	35.9

^a^Very High: HDI > =0.8

^b^High: 0.7 < =HDI < 0.8

^c^Medium: 0.55 < =HDI < 0.7

^d^Low: HDI < 0.55

*HDI* Human development index

**Table 5:** Frequency of countries based on income analytical category

	2013					2016				
	Frequency	Percent	Mean of mortality rate	Minimum mortality rate	Maximum mortality rate	Frequency	Percent	Mean of mortality rate	Minimum mortality rate	Maximum mortality rate
Very High^a^	38	33.0	7.079	2.8	23.4	34	30.1	6.003	2.7	13.4
Upper- Middle^b^	31	27.0	18.387	7.7	36.2	30	26.5	17.220	6.4	34.6
Lower- Middle^c^	30	26.1	20.813	10.5	29.1	34	30.1	19.874	9.7	30.1
Low^d^	16	13.9	27.569	13.6	35.0	15	13.3	28.360	15.9	35.9
Total	115	100.0	16.561	2.8	36.2	113	100.0	16.122	2.7	35.9

^a^Very High: GNI > = $12,736

^b^Upper- Middle: $4125 < =GNI < $12,736

^c^Lower- Middle: $1045 < =GNI < $4125

^d^Low: GNI < $1045

*GNI* Gross National Income

### Stepwise multivariate linear regression analysis

Stepwise multivariate linear regression analysis was initiated with eleven independent variables. The entry and exit criteria (the *P*-value of F-statistic) were set to 0.05 and 0.1, respectively (SPSS software default F-statistic *P*-values, Finlay (2012) [27]). In 2013, the final model consisted of five variables that explained the mortality rate (detailed regression coefficients are available in Additional file 1: Table S9). Specifically, income, safer vehicles, GINI index, life expectancy and safer road users were selected, represented in the following equation:

1 $$ Mortality=45.38-13.61\ast income-1.77\ast safe\ vehicle+0.17\ast GINI-17.91\ast life\ expectancy-1.52\ast safe\ user $$

As can be seen in Eq. (1), all the coefficients carry the expected signs. The R-squared (R^2^) value is the proportion of the variation of target variable which can be explained by its explanatory variables. The R^2^ value for Eq. (1) is 0.746, indicating a good model fit to the data (Table 6). Furthermore, Table 6 illustrates partial influence of the selected variables. Table 6 shows that 65% of the variation of the dependent variable is explained by income.

**Table 6:** Stepwise Multivariate Linear Regression: model summary (2013)

Model	R	R square	Adjusted R square	R square change
1	.804^a^	.646	.643	.646
2	.832^b^	.693	.688	.047
3	.849^c^	.720	.713	.027
4	.857^d^	.734	.724	.013
5	.864^e^	.746	.734	.012

^a^ Predictors: Constant, income 2013

^b^ Predictors: Constant, income 2013, safer vehicles 2013

^c^ Predictors: Constant, income 2013, safer vehicles 2013, GINI index 2013

^d^ Predictors: Constant, income 2013, safer vehicles 2013, GINI index 2013, life expectancy 2013

^e^ Predictors: Constant, income 2013, safer vehicles 2013, GINI index 2013, life expectancy 2013, safeuser2013

In 2016, the final model consists of three variables that explain mortality rate (detailed regression coefficients are shown in Additional file 1: Table S10). Specifically, income, GINI index and life expectancy were selected, represented in the following equation:

2 $$ Mortality=37.47-26.91\ast income+0.35\ast GINI-19.16\ast \mathrm{life}\ \mathrm{expectancy} $$

As can be seen in Eq. (2), all the coefficients carry the expected signs. The R^2^ value for Eq. (2) is 0.784, representing a good model fit to the data (Table 7). As can be seen in Table 7, 67% of the variation of the dependent variable is explained by income.

**Table 7:** Stepwise Multivariate Linear Regression: model summary (2016)

Model	R	R square	Adjusted R square	R square change
1	.821^a^	.674	.671	.674
2	.877^b^	.769	.765	.095
3	.885^c^	.784	.778	.015

^a^ Predictors: Constant, income 2016

^b^ Predictors: Constant, income 2016, GINI index 2016

^c^ Predictors: Constant, income 2016, GINI index 2016, life expectancy 2016

### Classification and regression trees

**Fig. 1:**
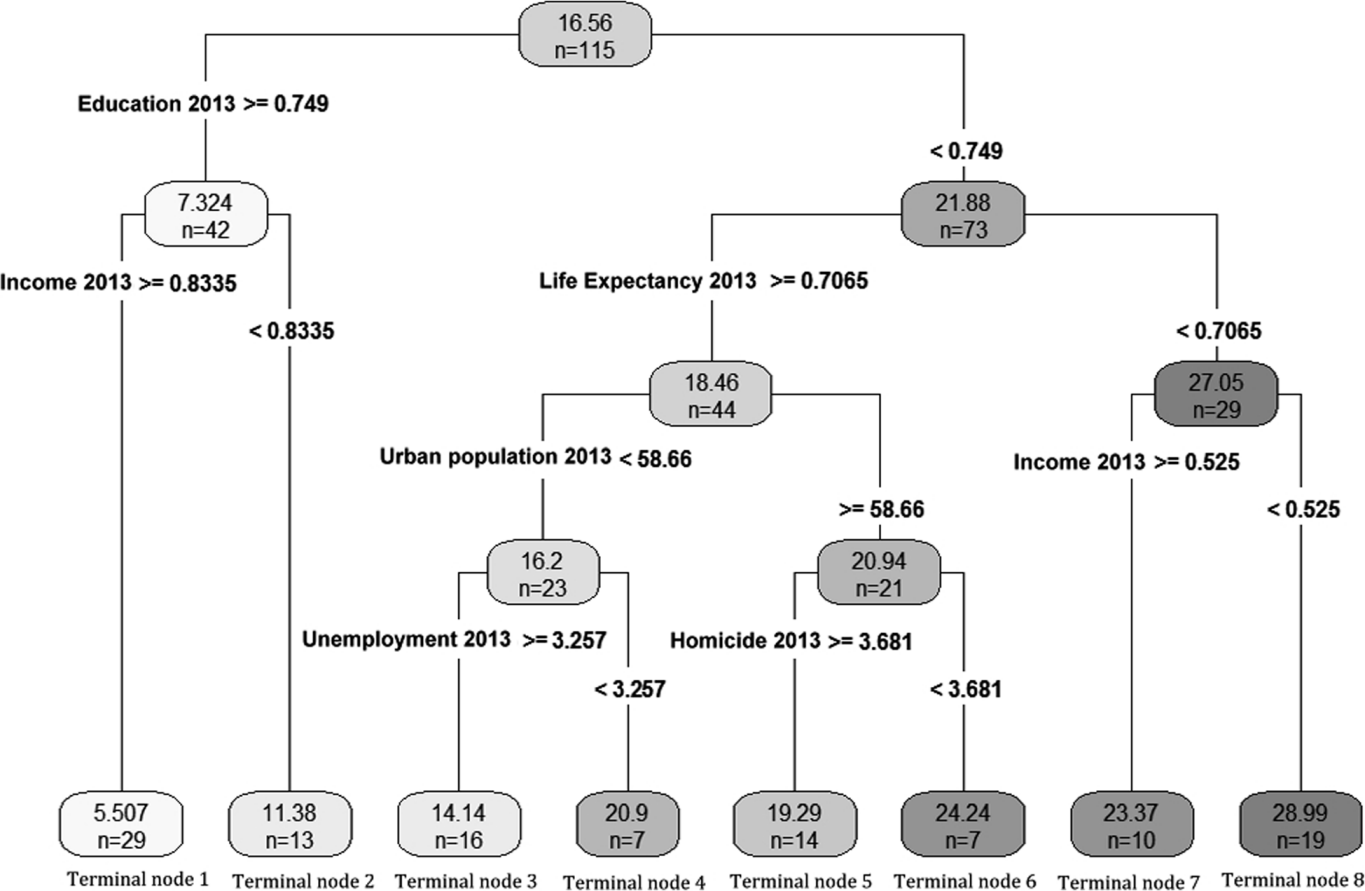
Optimal tree created by CART (2013). The number of countries and their associated mean of mortality rate are shown at each terminal node

Classification and regression trees analysis was performed using the eleven independent variables. Furthermore, in order to obtain an optimal model, a 10-fold cross-validation was carried out. In 2013, the result of CART is a tree with 7 non-terminal nodes and 8 terminal nodes (Fig. 1). From the 11 independent variables, CART used income, life expectancy, urban population, unemployment and homicide to build the optimal model.

**Fig. 2:**
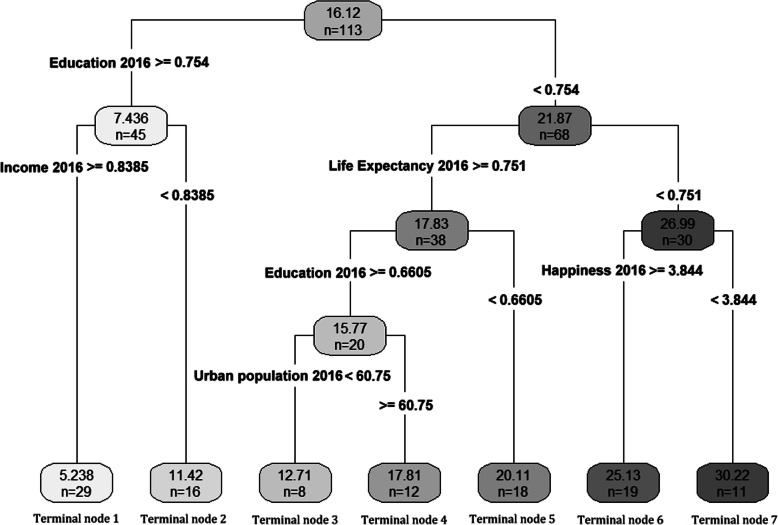
Optimal tree created by CART (2016). The number of countries and their associated mean of mortality rate are shown at each terminal node

In 2016, the result of CART is a tree with 6 non-terminal nodes and 7 terminal nodes (Fig. 2). CART used education, income, life expectancy, unemployment and happiness to build the optimal model. The rules and the mean of mortality rate from the final tree are available in Additional file 1: Tables S11 and S12. Moreover, the categorization of countries based on terminal nodes are shown in Additional files 2 and 3.

### Multivariate adaptive regression splines model

A first-order MARS was carried out, so the basis functions of the models consist of linear splines. In order to create smaller models during the pruning step, the GCV criterion was replaced with 10-fold cross-validation. In Table 8, the basis functions and their coefficients are shown in detail for 2013 data set. Then, the MARS prediction function is represented in the following equation:

$$ Y=16.99-1.2\ast BF1+0.09\ast BF2+191.51\ast BF3-437.70\ast BF4+225.58\ast BF5+47.72\ast BF6+36.05\ast BF7-66.44\ast BF8-36.37\ast BF9 $$

**Table 8:** Basis functions of the MARS and their coefficients (2013)

Variables	Basis Function	coefficients
(Intercept)		16.99
BF1	safer vehicles 2013	−1.2
BF2	h(urban population 2013–38.979)	0.09
BF3	h(education 2013–0.583)	191.51209
BF4	h(education 2013–0.623)	−437.70114
BF5	h(education 2013–0.654)	225.58727
BF6	h(income 2013–0.59)	47.72427
BF7	h(0.745 - income 2013)	36.05110
BF8	h(income 2013–0.745)	−66.43674
BF9	h(Life Expectancy 2013–0.613)	−36.36855

*BF* Basis function, h Hinge function

To illustrate the interpretation of MARS outcomes, consider the BF2 given in Table 8

$$ \mathrm{BF}2=\max \left(0,\mathrm{urban}\ \mathrm{population}-38.979\right)=\left\{\begin{array}{c}\ \mathrm{GINI}\ \mathrm{index}-38.979, if\ 38.979<\mathrm{urban}\ \mathrm{population}\\ {}0, otherwise\end{array}\right.. $$

Therefore, if the GINI index of a country is 40.979, then the MARS model predicts the mortality rate increase by 0.18 (i.e., 0.09*(40.979–38.979)); otherwise, if the GINI index of a country is less than 38.979, then GINI index has no effect on mortality rate.

As shown in Table 8, the MARS model contains 9 basis functions. It can be observed that five variables play an important role in determining mortality rate. These variables include safer vehicles, urban population, education, income, and life expectancy. The detailed impact of each basis function on mortality rate for 2013 data set are available in Additional file 1: Table S13. Based on Table S13 results, the mean of mortality rate of countries decreases when (safer vehicles > 0), (urban population > 38.979) and (life expectancy > 0.613). Furthermore, it can be inferred that the mean of mortality rate of countries decreased with increase of education and income.

Table 9 shows the basis functions and their coefficients for 2016 data set. The MARS prediction function is represented in the follow equation:

$$ Y=20.9-0.29\ast BF1+3.68\ast BF2-30.82\ast BF3+29.53\ast BF4-52.24\ast BF5. $$

**Table 9:** Basis functions of the MARS and their coefficients (2016)

Variables	Basis Function	coefficients
(Intercept)		20.9
BF1	h (45 - GINI index 2016)	−0.29
BF2	h(5.121 - happiness 2016)	3.68
BF3	h(education 2016–0.631)	−30.82
BF4	h(0.549 – income 2016)	29.53
BF5	h(life expectancy 2016–0.865)	−52.24

*BF* Basis function, h Hinge function

As can be seen in Table 9, the MARS model contains 5 basis functions. It can be observed that five variables play an important role in determining mortality rate. These variables are GINI index, happiness, education, income, and life expectancy. For more details on the impact of each basis function on mortality rate for 2016 data set refer to Additional file 1: Table S14. According to Table S14, when (happiness < 5.121) and (income < 0.549) we have an increase in mean of mortality rate of countries, while the mean of mortality rate of countries increases when (GINI< 45), (income < 0.549) and (life expectancy > 0.865).

### Changes in road traffic fatality

Comparing RTF data of 2013 and 2016 indicates that more than 20% of the change occurred in 18 countries, and more than 30% in 8 countries. Table 10 shows countries with more than 30% of change in RTF with corresponding growth rate in GINI index and HDI between these years.

**Table 10:** Countries with more than 30% change in RTF with correspondent growth rate in GINI index and HDI

Country	RTF rate 2013	RTF rate 2016	RTF growth rate	GINI index 2013	GINI index 2016	GINI index growth rate	HDI 2013	HDI 2016	HDI growth Rate
Iran	32.1	20.5	−36.14	37.4	40.0	6.95	0.784	0.796	1.53
Belarus	13.7	8.9	−35.04	26.6	25.3	−4.89	0.804	0.805	0.12
Bolivia	23.2	15.5	−33.19	47.6	44.6	−6.30	0.668	0.689	3.14
Macedonia	9.4	6.4	−31.91	36.2	35.6	−1.66	0.743	0.756	1.75
Kyrgyzstan	22.0	15.4	−30.00	28.8	26.8	−6.94	0.658	0.669	1.67
India	16.6	22.6	36.14	35.7	35.7	0.00	0.607	0.636	4.78
Turkey	8.9	12.3	38.20	40.2	41.9	4.23	0.771	0.787	2.07
Iceland	4.6	6.6	43.48	25.4	27.8	9.45	0.920	0.933	1.41

*RTF* Road traffic fatalities, *HDI* Human development index

### Prediction performance of the models

The indices described in the previous section are shown in Table 11 for each model. The values of indices indicated a good fit to the data for each model. It can be observed that CART has the best performance among other methods.

**Table 11:** Prediction performance measures of the models

Model	r		RMSE		MAE		RAE		R^**2**^	
	2013	2016	2013	2016	2013	2016	2013	2016	2013	2016
SMLR	0.86	0.88	4.62	4.27	3.21	3.11	0.40	0.39	0.75	0.78
CART	0.91	0.91	3.80	3.71	2.75	2.71	0.34	0.34	0.82	0.84
MARS	0.90	0.90	4.02	3.90	2.93	2.80	0.36	0.35	0.81	0.82

*r* correlation coefficient, *RMSE* Root mean squared error, *MAE* Mean absolute error, *RAE* Relative absolute error, *SMLR* Stepwise multivariate linear regression, *CART* Classification and regression trees, *MARS* Multivariate adaptive regression splines

Variable importance measure (VIM) is one of the useful outputs from the CART and MARS models, which reflects the effect of predictor variables on the model. Table 12 indicates the relative variable importance computed for the 9 independent variables in 2013 and 2016. There were considerable differences between the models regarding the importance of independent variables, which will be discussed in the next section.

**Table 12:** Importance of variables included in the CART and MARS model

Variable	Importance in CART		Importance in MARS	
	2013	2016	2013	2016
Education	21	21	100	100
Income	19	18	19.6	10.6
Life expectancy	17	17	38.7	15
Safer vehicles	14	13	5	unused
Happiness	12	5	unused	38.5
Homicide	10	12	unused	unused
Urban population	3	2	16.5	unused
Unemployment	2	1	unused	unused
GINI index	1	unused	unused	19.5
Safer road users	unused	11	unused	unused
Safer roads and mobility	unused	unused	unused	unused

*CART* Classification and regression trees, *MARS* Multivariate adaptive regression splines

## Discussion

This study aims to investigate the main factors related to road traffic fatality (RTF) around the world. Based on RSDI framework, we considered human performance, product performance and system performance. In this context, two main factors which play a critical role in RTF in both 2013 and 2016 years were HDI and GINI indices. Amongst the selected factors, the results showed that HDI was the main determinant, which was related to RTF. The results indicate that from 2013 to 2016, the mortality rate decreased along with GINI index, while HDI increased in this period (Additional file 1: Tables S4 to S6). In both years, HDI can explain more than 66% of RTF variations (Additional file 1: Tables S2 and S3).

To the best of our knowledge, there is scanty research similar in scope to the present study, and none as broad in data collection as our study. Most studies use panel data to determine factors which influence RTF. However, in these studies the number of indices or the number of countries is limited. Al-Haji found a strong relationship between HDI and RSDI and mentioned that by boosting the income of countries, more safer vehicles will be used and their investment on road infrastructure will be promoted [10, 28]. Yannis (2014) investigated 27 European countries between 1975 to 2011 and find out that by increasing Gross Domestic Product (GDP) (one of HDI components), RTF also increase in these countries [29]. Another investigation in 27 upper-middle-income countries in the three continents reveals that increasing GDP has a significant impact on RTF [30]. Bester (2001) found that passenger car ownership, HDI and the percentage of other vehicles had an impact on road traffic death and HDI was able to explain 53% of the variation of road death [31]. While in our study we found that HDI in SMLR was able to explain nearly 66% of the variation of mortality rate in 2013 and 72% in 2016. Melinder (2007) investigated 15 European countries and found a relationship amongst fatal death in road collisions, wealth and religion. She concluded that economic level has an impact on RTF to some point and other factors subsequently play a role [32]. A much broader study on 176 countries accounted for seven factors influencing road traffic injuries. These factors include income level of a country and some other factors that could be categorized under the subheading of the “safer road users” [33].

A total of 101 countries have valid data for both 2013 and 2016 (Additional file 4). Among these, the RTF of 18 countries changed more than ±20% (Additional file 5) and in 8 countries more than ±30% (Additional file 6). In this regard, by considering different variables related to RTF, only GINI index seemed to have a relationship with RTF in these 8 countries. RTF decreased dramatically in 5 countries in these three years more than 30%, namely Iran, Belarus, Bolivia, Macedonia and Kyrgyzstan. Except Iran, other four countries in these years experienced a reduction in their GINI index. On the other hand, RTF in three countries increased more than 30%, namely India, Turkey and Iceland. Except India, other two countries in these years are faced with an increasing rate in GINI index. Thus, the behavior analysis of Iran and India revealed different results from the other six countries. In the case of Iran (however not limited to Iran), this could be explained by inconsistency between the reported data of the country and WHO estimation. However, it should be mentioned that from 2006 to 2012 Iran faced a decrease in the absolute number of deaths (approximately 27%) [34]. On the other hand, the most recent available data for GINI index for India is attributed to the year 2011. Hence, as the most recent data have been used in this study (mentioned in methodology section), for GINI index in 2013 and 2016, there was no difference between these two years. These results show the importance of GINI index mainly for countries with more than 30% changes. However, there is a need to investigate the relation between RTF and GINI index in a long run.

All the eight countries subject to analysis, except Iceland (very high HDI rank), had the HDI between 0.6 and 0.8 (high and medium HDI rank), which experienced the most radical changes. It is postulated that these countries might be more fragile to GINI index changes.

In this study, three different procedures for analyzing data in 115 countries were applied in 2013 and for 2016, 113 countries were assessed. As HDI is the main factor related to RTF, three components of HDI (income, education and life expectancy) were considered alongside other factors. By using SMLR, income is the main factor related to RTF in both years and it could explain 64 and 67% of RTF variation in 2013 and 2016, respectively. It is noticeable that when income is the main factor to explain RTF, GINI index also affects our output in both years. In 2013, safer vehicles, life expectancy and safer road users were also related to RTF. However, these factors were not present in 2016.

Other two procedures have better explanation than SMLR. CART and MARS models reveal that education is the main factor related to RTF among other factors. Variable importance table shows that for both years education, income and life expectancy (HDI components) are the main factors related to RTF respectively. For 2013, the cut-off point of education index is 0.749 and countries which have higher education index have lower RTF. In this context, countries with income index of more than 0.8335 have the lowest RTF rate (29 countries). On the other hand, for countries with education index of lower than 0.749, life expectancy has a significant relationship with RTF. Life expectancy cut-off point for these countries is 0.7065 and countries with higher life expectancy have lower rate of RTF (Additional file 2). These results have been approximately confirmed by MARS model, where education index, income index and life expectancy index have the highest coefficient than other factors. This trend can also be seen in 2016. In this year, for CART model, education index cut-off point is 0.754 and countries with income index of higher than 0.8385 have the lowest RTF (29 countries). On the other hand, countries with education index of lower than 0.754, life expectancy with the cut-off point of more than 0.751 have lower RTF than other countries in this category. In MARS model, these three variables also have the highest impact on RTF (Additional file 3).

In addition, based on CART and MARS models, legislative factors do not have valid relationships with RTF for both years. However, regarding CART model and factor importance table, safer vehicles could be related to RTF if HDI components are not considered.

By considering World Bank classification 2016 [35] in the CART model for both 2013 and 2016, countries with the least RTF fatality (29 countries for both years), are high-income countries (GNI of more than $12,736) with very high HDI rank (HDI of more than 0.8). On the other hand, countries with the highest rate of RTF (19 countries for 2013 and 11 countries for 2016) are mostly low-income (GNI of $1045 or less) and low developed (HDI less 0.55). Therefore, based on the above discussion, we can conclude that for both groups, the level of education is the most important factor related to RTF and at the next level, income plays a central role for both high- and low-income countries. For other countries, although it can be seen that this trend is mainly valid (higher rate of HDI and education associated with less RTF), it can be concluded that by enhancing education rate and the income might reduce the RTF rate.

In 2013, among 38 high-income countries, only four countries (Republic of Korea, Chile, Uruguay and Brazil) had an RTF of more than one-third of the global RTF range (maximum (RTF) - minimum (RTF)). However, in 2016, only Chile and Uruguay have had an RTF of more than 12 per 100,000 population among the high-income countries. Republic of Korea reduced its RTF from 12 to 9.8 per 100,000 population between 2013 and 2016, and in 2016 Brazil was not ranked as a high-income country and was downgraded to a middle-high income country. On the other hand, except Bangladesh and Nepal (with RTF 13.6 and 17 per 100,000 population, respectively) other low-income countries had an RTF of more than 25 per 100,000 population. In 2016, Bangladesh was promoted to a middle-income country and Nepal had an RTF of lower than 16 per 100,000 population. Other low-income countries had an RTF of more than 23 per 100,000 population. Regarding this data, there is a need to consider these exceptions and investigate the cause of a different behavior in the mentioned countries than other countries in their category. For example, Nepal can be a good case study among low-income countries, which reduced its RTF with limited resources.

## Limitations

This is a cross-sectional study and there is a need for a longitudinal study to assess the impact of different factors on mortality rate and investigate the casual relations among HDI components with RTF rate. Although we tried to consider nearly 180 countries, missing data reduced our cases to approximately 115 countries. Besides, however we have considered happiness and homicide rate for behavioral dimension of RSDI, we did not consider some individual-level variables, such as personality or personal ability. Finally, some important factors such as road network size of countries or their investment in road industry were not accessible for most countries in 2013 and 2016; hence, we did not consider these factors.

## Conclusion

To sum up, although legislative factors, urban population and happiness can play important roles in the prediction of RTF for some countries, we found that HDI can be the core predictor for 2013 and 2016 in most countries. Considering HDI components gives us a wider view about factors related to RTF. By comparing SMLR, CART, and MARS models, it can be seen that CART provides a better explanation than others with R^2^ of nearly 83% for both years. Based on CART results, education can play a central role in decreasing RTF in countries and by investing on public education via school, countries could reduce their road fatality. For countries with a high rate of education index, income plays an important role too. This can be due to their investigation on road safety and using better vehicles. For countries with lower education, better medical care can reduce their vulnerability to RTF. In addition, high-income countries mostly have the lowest number of RTF and low-income countries have the highest rate of RTF. As a result, policymakers should consider RTF as a socio-economic problem which requires both social and economic solutions.

## Supplementary information

**Additional file 1.** Details of methodology.

**Additional file 2.** Categorization of countries based on CART result in 2013.

**Additional file 3.** Categorization of countries based on CART result in 2016.

**Additional file 4.** Countries which have valid data for both 2013 and 2016.

**Additional file 5.** Countries changed more than ±20% in RTF.

**Additional file 6.** Countries changed more than ±30% in RTF.

## Data Availability

The datasets used and/or analyzed during the study are available from the corresponding author on reasonable request.

## Abbreviations

RTF: Road traffic fatalities
RSDI: Road Safety Development Index
CART: Classification and Regression Trees
MARS: Multivariate Adaptive Regression Splines
HDI: Human Development Index
WHO: World Health Organization
SMLR: Stepwise Multivariate Linear Regression
LSD: Least Squared Deviation
BF: basis function
GCV: Generalized Cross-Validation criterion
RMSE: Root Mean-Squared Error
MAE: Mean Absolute Error
RAE: Relative Absolute Error
GDP: Gross Domestic Product
