# Supplementary material for: Social, economic, and legislative factors and global road traffic fatalities
Source: BMC Public Health. 2020 Sep 17;20:1413. doi: 10.1186/s12889-020-09491-x (PMC7646406; doi:10.1186/s12889-020-09491-x)
Supplement: Supplementary file 1 — Additional file 1. Details of methodology. [file 12889_2020_9491_MOESM1_ESM.docx]

**Supplementary appendix**

**Supplement to:** Mohammad Reza Rahmanian Haghighi, Mohammad Sayari , Sulmaz Ghahramani, Kamran Bagheri Lankarani.

Social, economic, and legislative factors and global road traffic fatalities.

**Table of Contents**

**TABLES**

#

[**Table S1. Safe System (factors and their indices).** 3](#_Toc36324083)

[**Table S2. Stepwise multivariate linear regression: model summary (2013).** 4](#_Toc36324084)

[**Table S3. Stepwise multivariate linear regression: model summary (2016).** 4](#_Toc36324085)

[**Table S4. Impact of BF1, BF2, BF8 and BF9 on mortality rate in initial model (2013).** 5](#_Toc36324086)

[**Table S5. Impact of BF3 to BF7 on mortality rate in initial model (2013).** 6](#_Toc36324087)

[**Table S6. Impact of BF1, BF2, BF3 and BF4 on mortality rate in initial model (2016).** 7](#_Toc36324088)

[**Table S7. Prediction performance measures of the initial models.** 8](#_Toc36324089)

[**Table S8. Importance of variables included in the CART and MARS model.** 8](#_Toc36324090)

[**Table S9. Stepwise multivariate linear regression: Coefficients (2013).** 9](#_Toc36324091)

[**Table S10. Stepwise multivariate linear regression: Coefficients (2016).** 9](#_Toc36324092)

[**Table S11. CART pruning rules (2013).** 10](#_Toc36324093)

[**Table S12. CART pruning rules (2016).** 11](#_Toc36324094)

[**Table S13. Impact of each basis function on mortality rate (2013).** 12](#_Toc36324095)

[**Table S14. Impact of each basis function on mortality rate (2016).** 13](#_Toc36324096)

**Table S1. Safe system (factors and their indices).**

| Safer roads and mobility | Safer vehicles | Safer road users |
| --- | --- | --- |
| Formal audits required for new road construction projects (2013) | Frontal impact standard (for both years) | National speed limit law (for both years) |
| Regular inspections of existing road infrastructure (2013) | Electronic stability control (for both years) | National drink–driving law (for both years) |
| Policies to promote walking or cycling (2013) | Pedestrian protection (for both years) | National motorcycle helmet law (for both years) |
| Policies to encourage investment in public transport (2013) | Motorcycle anti-lock braking system (2016) | National seat-belt law (for both years) |
| Policies to separate road users and protect Vulnerable Road Users (VRUs) (2013) |  | National child restraint law (for both years) |
| Audits or star rating required for new road  Infrastructure (2016) |  | National law on mobile phone use while driving (for both years) |
| Design standards for the safety of pedestrians /  Cyclists (2016) |  | National drug-driving law (for both years) |
| Inspections / star rating of existing roads (2016) |  |  |
| Investments to upgrade high risk locations (2016) |  |  |
| Policies & investment in urban public transport (2016) |  |  |

# **Results of Initial model (model with presence of HDI)**

**Table S2. Stepwise multivariate linear regression: model summary (2013).**

| Model | R | R Square | Adjusted R Square | Std. Error of the Estimate | R Square Change |
| --- | --- | --- | --- | --- | --- |
| 1 | .819^a^ | .671 | .668 | 5.3144 | .671 |
| 2 | .842^b^ | .710 | .705 | 5.0104 | .039 |
| 3 | .853^c^ | .728 | .721 | 4.8696 | .019 |
| 4 | .862^d^ | .742 | .733 | 4.7643 | .014 |

1. Predictors: Constant, HDI2013
2. Predictors: Constant, HDI2013, safer vehicles 2013
3. Predictors: Constant, HDI2013, safer vehicles 2013, GINI 2013
4. Predictors: Constant, HDI2013, safer vehicles 2013, GINI 2013, safer road users 2013

HDI Human development index

**Table S3. Stepwise multivariate linear regression: model summary (2016).**

| Model | R | R Square | Adjusted  R Square | Std. Error  of the  Estimate | R Square  Change |
| --- | --- | --- | --- | --- | --- |
| 1 | .850^a^ | .722 | .720 | 4.8723 | .722 |
| 2 | .885^b^ | .783 | .779 | 4.3315 | .060 |

1. Predictors: Constant, HDI 2016
2. Predictors: Constant, HDI 2016, GINI 2016

HDI Human development index

**Table S4. Impact of BF1, BF2, BF8 and BF9 on mortality rate in initial model (2013).**

| **GINI** | | | | | |
| --- | --- | --- | --- | --- | --- |
| **Fold** | **Rule** | **Role in prediction function** | **Number of countries** | **Mean of mortality rate** | **Mean of predicted mortality rate** |
| 1 | GINI<=30.8 | 0 | 22 | 10.938 | 10.969 |
| 2 | GINI>30.8 | 0.25* BF1 | 93 | 18.044 | 18.036 |
| **Unemployment** | | | | | |
| **Fold** | **Rule** | **Role in prediction function** | **Number of countries** | **Mean of mortality rate** | **Mean of predicted mortality rate** |
| 1 | Unemployment<=10.36 | 0 | 88 | 17.093 | 17.257 |
| 2 | Unemployment>10.36 | -0.22* BF2 | 27 | 14.826 | 14.291 |
| **Safer roads and mobility** | | | | | |
| **Fold** | **Rule** | **Role in prediction function** | **Number of countries** | **Mean of mortality rate** | **Mean of predicted mortality rate** |
| 1 | Safer roads and mobility <=4 | 0 | 89 | 19.049 | 19.049 |
| 2 | Safer roads and mobility >4 | -2.46* BF7 | 26 | 8.042 | 8.042 |
| **Safer road users** | | | | | |
| **Fold** | **Rule** | **Role in prediction function** | **Number of countries** | **Mean of mortality rate** | **Mean of predicted mortality rate** |
| 1 | Safer road users <=6 | 0 | 53 | 22.726 | 22.726 |
| 2 | Safer road users >6 | -3.1* BF8 | 62 | 11.290 | 11.290 |
| **Total** | | | 115 | 16.56 | 16.56 |

BF Basis function

As can be seen in Table S4, when (GINI>30.8) we have an increase in mean of mortality rate, but when (Unemployment>10.36), (Safer roads and mobility >4) and (Safer road users >6) a decreasing trend mortality rate was observed.

**Table S5. Impact of BF3 to BF7 on mortality rate in initial model (2013).**

| HDI | | | | | |
| --- | --- | --- | --- | --- | --- |
| **Fold** | **Rule** | **prediction function** | **Number of countries** | **Mean of Mortality rate** | **Mean of predicted Mortality rate** |
| 1 | HDI <=0.618 | 62.28* BF4 | 33 | 25.261 | 25.305 |
| 2 | 0.618< HDI < 0.741 | 106.68*BF3+62.28* BF4 | 24 | 20.733 | 20.521 |
| 3 | HDI=0.741 | 0 | 1 | 23.8 | 21.838 |
| 4 | 0.741< HDI <= 0.759 | 106.68*BF3+62.28* BF4  -405.88*BF5 | 6 | 13.917 | 17.925 |
| 5 | HDI >0.759 | 106.68*BF3+62.28* BF4  -405.88*BF5+251.99*BF6 | 51 | 9.137 | 8.775 |
| Total | | | 115 | 16.56 | 16.56 |

HDI Human development index, BF Basis function

From the results in Table S5, it can be inferred that the Mortality rate of countries decreased with increase of HDI.

**Table S6. Impact of BF1, BF2, BF3 and BF4 on mortality rate in initial model (2016).**

| **GINI** | | | | | |
| --- | --- | --- | --- | --- | --- |
| **Fold** | **Rule** | **Prediction function** | **Number of countries** | **Mean of mortality rate** | **Mean of predicted mortality rate** |
| 1 | GINI<=31.8 | 0 | 27 | 8.17 | 8.50 |
| 2 | GINI>31.8 | 0.31* BF1 | 86 | 18.61 | 18.51 |
| **Happiness** | | | | | |
| **Fold** | **Rule** | **Prediction function** | **Number of countries** | **Mean of mortality rate** | **Mean of predicted mortality rate** |
| 1 | Happiness >=4.875 | 0 | 72 | 11.63 | 11.54 |
| 2 | Happiness <4.875 | 4.24* BF2 | 41 | 24.00 | 24.16 |
| **HDI** | | | | | |
| **Fold** | **Rule** | **Prediction function** | **Number of countries** | **Mean of mortality rate** | **Mean of predicted mortality rate** |
| 1 | HDI<0.746 | 35.53*BF3 | 51 | 23.49 | 23.41 |
| 2 | HDI=0.746 | 0 | 1 | 13.70 | 17.20 |
| 3 | HDI >0.746 | -62.32*BF4 | 61 | 10.00 | 10.00 |
| Total | | | 113 | 16.12 | 16.12 |

HDI Human development index, BF Basis function

As can be seen in Table S6, when (GINI>31.8), (happiness <4.875) and (HDI<0.746) we have an increase in mean of mortality rate, but when (HDI >0.746) a decreasing trend mortality rate was observed.

**Table S7. Prediction performance measures of the initial models.**

|  | **r** | | **RMSE** | | **MAE** | | **RAE** | | **R^2^** | |
| --- | --- | --- | --- | --- | --- | --- | --- | --- | --- | --- |
| **Model** | **2013** | **2016** | **2013** | **2016** | **2013** | **2016** | **2013** | **2016** | **2013** | **2016** |
| SMLR | 0.86 | 0.88 | 4.67 | 4.27 | 3.30 | 3.11 | 0.41 | 0.39 | 0.74 | 0.78 |
| CART | 0.87 | 0.92 | 4.38 | 3.40 | 3.08 | 2.59 | 0.40 | 0.33 | 0.77 | 0.86 |
| MARS | 0.89 | 0.90 | 4.16 | 3.96 | 3.28 | 2.80 | 0.38 | 0.35 | 0.79 | 0.81 |

r Correlation coefficient, RMSE Root mean squared error, MAE Mean absolute error, RAE Relative absolute error, SMLR Stepwise multivariate linear regression, CART Classification and regression trees, MARS Multivariate adaptive regression splines

**Table S8. Importance of variables included in the CART and MARS model.**

|  | **Importance**  **in CART** | | **Importance**  **in MARS** | |
| --- | --- | --- | --- | --- |
| **Variable** | **2013** | **2016** | **2013** | **2016** |
| HDI | 29 | 24 | 100 | 100 |
| Happiness | 16 | 16 | unused | 12.3 |
| Safer road users | 11 | 15 | 7.8 | unused |
| Safer vehicles | 18 | 14 | unused | unused |
| Urban population | 12 | 13 | unused | unused |
| Homicide | 12 | 12 | unused | unused |
| GINI | 01 | 03 | 16.0 | 24.2 |
| Unemployment | 01 | 02 | 3.6 | unused |
| Safer roads and mobility | unused | 01 | unused | unused |

HDI Human development index

# **Results of model with HDI components (education, income and life expectancy)**

**Table S9. Stepwise multivariate linear regression: coefficients (2013).**

| Model | Unstandardized coefficients | | Standardized coefficients | t | Sig. |
| --- | --- | --- | --- | --- | --- |
|  | B | Std. Error | Beta |  |  |
| (Constant) | 45.374 | 5.568 |  | 8.149 | .000 |
| Income 2013 | -13.609 | 6.116 | -.258 | -2.225 | .028 |
| Safer vehicles 2013 | -1.763 | .486 | -.265 | -3.628 | .000 |
| GINI 2013 | .168 | .063 | .144 | 2.657 | .009 |
| Life expectancy 2013 | -17.907 | 7.078 | -.243 | -2.530 | .013 |
| Safer road users 2013 | -1.485 | .644 | -.147 | -2.305 | .023 |

**Table S10. Stepwise multivariate linear regression: coefficients (2016).**

| Model | Unstandardized coefficients | | Standardized coefficients | t | Sig. |
| --- | --- | --- | --- | --- | --- |
|  | B | Std. Error | Beta |  |  |
| (Constant) | 37.474 | 4.913 |  | 7.628 | .000 |
| Income 2016 | -26.914 | 4.525 | -.517 | -5.948 | .000 |
| GINI 2016 | .353 | .061 | .287 | 5.834 | .000 |
| Life expectancy 2016 | -19.160 | 7.073 | -.245 | -2.709 | .008 |

**CART pruning rules and the mean of mortality rate of each terminal node**

**Table S11. CART pruning rules (2013).**

| Terminal node | Rule | Number of countries | Mean of mortality rate | Mean of predicted mortality rate |
| --- | --- | --- | --- | --- |
| 1 | If (education>= 0.749) & (income >= 0.8335) | 29 | 5.507 | 5.5068 |
| 2 | If (education>= 0.749) & (income < 0.8335) | 13 | 11.377 | 11.3769 |
| 3 | If (education< 0.749) & (life expectancy >= 0.7065) & (urban population< 58.66) & (unemployment>=3.257) | 16 | 14.144 | 14.1437 |
| 4 | If (education< 0.749) & (life expectancy >= 0.7065)  & (urban population< 58.66) & (unemployment< 3.257) | 7 | 20.900 | 20.900 |
| 5 | If (education < 0.749) & (life expectancy >= 0.7065)  & (urban population>=58.66) & (homicide>=3.681) | 14 | 19.293 | 19.2928 |
| 6 | If (education< 0.749) & (life expectancy >=0.7065)  & (urban population>= 58.66) & (homicide< 3.681) | 7 | 24.243 | 24.2428 |
| 7 | If (education < 0.749) & (life expectancy < 0.7065)  & (income >= 0.525) | 10 | 23.370 | 23.370 |
| 8 | If (education < 0.749) & (life expectancy < 0.7065)  & (income < 0.525) | 19 | 28.989 | 28.989 |
| Total | | 115 | 16.561 | 16.561 |

**Table S12. CART pruning rules (2016).**

| Terminal node | Rule | Number of countries | Mean of mortality rate | Mean of predicted mortality rate |
| --- | --- | --- | --- | --- |
| 1 | If (education>= 0.754) & (income >= 0.8385) | 29 | 5.238 | 5.932 |
| 2 | If (education>= 0.754) & (income < 0.8385) | 16 | 11.42 | 11.410 |
| 3 | If (education< 0.754) & (life Expectancy >= 0.751)  & (education>=0.6605) & (urban population >=60.75) | 8 | 12.7125 | 12.7125 |
| 4 | If (education< 0.754) & (life expectancy >= 0.751) & (education>=0.6605) & (urban population < 60.75) | 12 | 17.808 | 17.8083 |
| 5 | If (education< 0.754) & (life expectancy >= 0.751)  & (education<0.6605) | 18 | 20.111 | 20.111 |
| 6 | If (education < 0.754) & (life expectancy < 0.751)  & (happiness >= 3.844) | 19 | 25.126 | 25.1263 |
| 7 | If (education < 0.754) & (life Expectancy < 0.751)  & (happiness < 3.844) | 11 | 30.218 | 30.218 |
| Total | | 113 | 16.122 | 16.122 |

**Table S13. Impact of each basis function on mortality rate (2013).**

| **Safer vehicles 2013** | | | | | |
| --- | --- | --- | --- | --- | --- |
| **Fold** | **Rule** | **prediction function** | **Number of countries** | **Mean of Mortality rate** | **Mean of predicted mortality rate** |
| 1 | Safer vehicles =0 | 0 | 70 | 21.451 | 21.674 |
| 2 | Safer vehicles >0 | -1.2 * BF1 | 45 | 8.953 | 8.606 |
| **urban population 2013** | | | | | |
| **Fold** | **Rule** | **prediction function** | **Number of countries** | **Mean of Mortality rate** | **Mean of predicted mortality rate** |
| 1 | Urban population <=38.979 | 0 | 23 | 24.087 | 24.142 |
| 2 | Urban population >38.979 | 0.09* BF2 | 92 | 14.68 | 14.67 |

| **Education 2013** | | | | | |
| --- | --- | --- | --- | --- | --- |
| **Fold** | **Rule** | **prediction function** | **Number of countries** | **Mean of Mortality rate** | **Mean of predicted mortality rate** |
| 1 | Education <=0.583 | 0 | 38 | 24.32 | 24.34 |
| 2 | 0.583< Education < 0.623 | 191.51*BF3 | 5 | 25.580 | 24.17 |
| 3 | 0.623< Education <= 0.654 | 191.51*BF3-437.70* BF4 | 5 | 19.960 | 22.38 |
| 4 | Education >0.654 | 191.51*BF3-437.70* BF4+225.58*BF5 | 67 | 11.239 | 11.15 |

| **Income 2013** | | | | | |
| --- | --- | --- | --- | --- | --- |
| **Fold** | **Rule** | **prediction function** | **Number of countries** | **Mean of Mortality rate** | **Mean of predicted mortality rate** |
| 1 | Income <=0.59 | 36.05* BF7 | 33 | 25.19 | 25.14 |
| 2 | 0.59< Income =< 0.745 | 47.724*BF6+36.05* BF7 | 30 | 19.1 | 18.86 |
| 3 | Income >0.745 | 47.724*BF6-66.43* BF8 | 52 | 9.62 | 9.79 |

| **Life Expectancy 2013** | | | | | |
| --- | --- | --- | --- | --- | --- |
| **Fold** | **Rule** | **prediction function** | **Number of countries** | **Mean of Mortality rate** | **Mean of predicted mortality rate** |
| 1 | Life Expectancy <=0.613 | 0 | 13 | 27.53 | 28.24 |
| 2 | Life Expectancy >0.613 | -36.37* BF9 | 102 | 15.16 | 15.07 |
| **Total** | | | 115 | 16.56 | 16.56 |

BF Basis function

**Table S14. Impact of each basis function on mortality rate (2016).**

| **GINI 2016** | | | | | |
| --- | --- | --- | --- | --- | --- |
| **Fold** | **Rule** | **prediction function** | **Number of countries** | **Mean of Mortality rate** | **Mean of predicted mortality rate** |
| 1 | GINI>=45 | 0 | 17 | 23.435 | 22.017 |
| 2 | GINI<45 | -0.29* BF1 | 96 | 18.827 | 15.078 |
| **Happiness 2016** | | | | | |
| **Fold** | **Rule** | **Role in prediction function** | **Number of countries** | **Mean of Mortality rate** | **Mean of predicted mortality rate** |
| 1 | Happiness >=5.121 | 0 | 67 | 11.134 | 11.185 |
| 2 | Happiness < 5.121 | 3.68* BF2 | 46 | 23.387 | 23.313 |
| **Education 2016** | | | | | |
| **Fold** | **Rule** | **Role in prediction function** | **Number of countries** | **Mean of Mortality rate** | **Mean of predicted mortality rate** |
| 1 | Education <=0.631 | 0 | 42 | 24.25 | 24.79 |
| 2 | Education >0.631 | -30.82* BF3 | 71 | 11.314 | 10.996 |
| **Income 2016** | | | | | |
| **Fold** | **Rule** | **Role in prediction function** | **Number of countries** | **Mean of mortality rate** | **Mean of predicted mortality rate** |
| 1 | Income >=0.549 | 0 | 87 | 13.047 | 13.161 |
| 2 | Income <0.549 | 29.53* BF4 | 26 | 26.412 | 26.032 |
| **Life Expectancy 2016** | | | | | |
| **Fold** | **Rule** | **Role in prediction function** | **Number of countries** | **Mean of Mortality rate** | **Mean of predicted mortality rate** |
| 1 | Life Expectancy <=0.865 | 0 | 73 | 20.853 | 20.684 |
| 2 | Life Expectancy >0.865 | -52.24* BF5 | 40 | 7.488 | 7.796 |
| **Total** | | | 113 | 16.122 | 16.122 |

BF Basis function
